# Integrated analysis identified prognostic microRNAs in breast cancer

**DOI:** 10.1186/s12885-022-10242-x

**Published:** 2022-11-12

**Authors:** Cong Shang, Qingyan Chen, Fuqiang Zu, Weidong Ren

**Affiliations:** 1 Department of Ultrasound, Shengjing Hospital of China Medical University, Shenyang, 110004 Liaoning China; 2 Shengjing Hospital of China Medical University, Shenyang, 110004 Liaoning China

**Keywords:** Breast cancer, Risk model, Nomogram, miR-127, Tumor progression

## Abstract

**Background:**

MicroRNAs (miRNAs) play pivotal roles in the development and progression of breast cancer (BC). In this study, we attempted to identify miRNAs associated with BC prognosis and progression via integrated analysis.

**Methods:**

We first screened 83 differentially expressed miRNAs (DEMs) in 1249 BC samples and 151 normal samples. We then validated their roles in expression and prognosis of BC, identified two survival-related DEMs, and established a risk model. The prediction efficiency was assessed in both the training and validation groups. Tissue and cell experiments were conducted to verify the regulatory effects of miR-127 in BC.

**Results:**

The ROC curve indicated good prediction ability with 1-, 3-, and 5-year survival rates of 0.73, 0.72, and 0.72, respectively. Moreover, hsa-miR-127 was found to be an independent prognostic factor of BC. Functional analyses revealed that it is involved in various cancer pathways such as the PI3K-Akt and p53 pathways. miR-127 expression was down-regulated in both BC tissues and cell lines. The knockdown of miR-127 substantially increased, whereas overexpression decreased BC cell proliferation, invasion, and migration. This effect of miR-127 was consistent with its tumorigenic ability and tumor volume in nude mice.

**Conclusions:**

These findings indicate that low expression of miR-127 contributes to BC migration, invasion, and tumorigenesis and that it can be a therapeutic target and prognostic biomarker for BC.

**Supplementary Information:**

The online version contains supplementary material available at 10.1186/s12885-022-10242-x.

## Background

Breast cancer (BC) is the most common malignant tumor in women, with the highest incidence and second highest mortality worldwide [1]. Approximately169,000 BC patients are diagnosed each year, with a quarter of the deaths occurring in China [2]. Although advances in treatment have improved the overall survival (OS) of BC patients, 30% of patients die from recurrence and metastasis [3]. Therefore, determining the underlying mechanism of BC invasion and metastasis is urgently needed.

In the human genome, only 2% of genes are encoded in messenger RNA (mRNA), and more than 98% are transcribed in non-coding RNA (ncRNA), indicating the importance of ncRNA in protein production [4, 5]. This phenomenon has also been observed in cancer tumorigenesis, progression, and metastasis [6]. MicroRNAs(miRNAs) are highly conserved short non-coding RNAs–20–24 nucleotides in length [7, 8]. Although miRNAs do not possess an open reading framework (ORF), they can assemble into an RNA-induced silencing complex (RISC) and target complementary mRNA sequences to inhibit their translation or degradation [7]. Up to 60% of mRNAs contain at least one miRNA complementary sequence, indicating that miRNAs strongly regulate mRNAs [9].

Additionally, the disturbance of miRNAs has great significance in cellular processes, tumor invasion, angiogenesis, and metastasis in BC [10, 11]. miRNAs are inhibited by inactivating oncogenes such as miR-10b, miR-146a, miR-181, miR-24, miR-29a, and miR-520c [12, 13]. Some miRNAs can promote BC progressions, such as miR-30, miR-31, miR-126, miR-146a, miR-206, and miR-503 [14–17]. Some miRNAs are considered therapeutic targets for BC, including miR-30c, miR-187, and miR-339-5p [18, 19]. MiRNAs are also used to evaluate BC diagnosis and prognosis because they are stable, easy to detect, and highly tissue-specific. For example, miR-148a and miR-335 can be used as diagnostic markers [20, 21], and miR-30c, miR-187, and miR-339-5p can predict the therapeutic efficacy of chemotherapy [19, 21]. These studies suggest an imperative role for miRNAs in BC progression. Therefore, it is necessary to explore miRNA disorders in BC.

In the present study, we comprehensively evaluated miRNA disorders and their role in prognosis. A nomogram is a simple and accurate model based on patient survival in every variable, which allows clinicians to quickly evaluate survival outcomes and make decisions. We developed a miRNA-based prognostic model based on these advantages and evaluated its efficiency in the training and validation groups. In addition, we verified miR-127 roles in BC cell proliferation, migration, invasion, and tumorigenicity through Vitro experiments. These findings shed light on novel treatment strategies for BC and provide a therapeutic target and prognostic biomarker for patients with BC.

## Materials and methods

### Data selection and process

To avoid bias caused by a single database, we systematically evaluated differentially expressed miRNAs (DEMs) by integrating RNA-Seq data of 1,400 BC samples obtained from the Cancer Genome Atlas(TCGA) [22] and Gene Expression Omnibus (GEO) databases [23]. The raw count data of TCGA_BRCA dataset were downloaded from the Genetic Disease Control (GDC) database, including 1104 breast cancer samples and 113 normal samples. The expression of miRNAs was extracted from raw data, unified, and normalized using the "limma" package [24]. Then, the DEMs was identified using the "DESeq2" package [25] with the threshold of |log2FC|≥ 0.5, *P*-value < 0.05. For the GEO database of BC, samples were systematically screened according to the following inclusion criteria:(1) human BC tissue,(2)the data type of miRNA expression was an array, and (3) both tumor and non-tumor samples were greater than 10. Finally, two GEO datasets were included, the GSE38167 dataset consisting of 67 samples with 44 BC samples and 23 normal samples, and the GSE45666 dataset consisting of 116 samples, including 101 BC samples and 15 normal samples. The matrix data were obtained, normalized, standardized, and subjected to DEMs using the cut-off criteria mentioned above. The annotation of miRNAs in TCGA and GEO was limited. So, we expanded the cut-off criteria of DEMs with |log2FC|≥ 0.5 rather than |log2FC|≥ 1 to obtain more comprehensive miRNA data. Common DEMs in the three datasets were screened using a Venn diagram [26] and selected for further analysis.

### Establishing a prognostic model

To explore the roles of DEMs in BC prognosis, we extracted data on survival time and survival status from TCGA_BRCA dataset and removed samples without OS or survival time of fewer than 30 days. Hazard ratios (HR) and 95% confidence intervals (CI) for each gene were estimated using univariate and multivariate COX regression analyses. Only miRNAs with *P* < 0.05 were identified as prognostic miRNAs. Next, we estimated the prognostic risk score for each patient using the following formula: risk score = X1α1 + X2α2 + X3α3 + … + Xnαn. Patients were divided into high- and low-risk groups based on the median risk score. Subsequently, the prognostic miRNAs were used to construct a nomogram risk model. Calibration curves at 3 and 5y were used to evaluate the reliability of the nomogram model for prognostic prediction. Kaplan–Meier (KM) analysis was used to estimate the difference in OS between the high-risk and low-risk groups. Then, we assessed the prediction performance of the risk model using receiver operating characteristic (ROC) curves at 1, 3, and 5 y and computed their area under the curve (AUC) values in the three groups. Moreover, we validated the prognosis of miR-127-5p in the METABRIC database and detected its survival roles in the ER-positive group, HER2 negative group, TNBC, lymph node positive group, and luminal A subtypes. To reveal the characteristic of hsa-miR-127 and hsa-miR-340 in different BC subgroups, we obtained PAM50 data from TCGA. PAM50 was a widely accepted gene test and divided into 5 subtypes according to BC genome phenotype, including LumA (Luminal A), LumB, Her2, Basel, Normal. In clinical application, Basel and Normal subtypes were regarded as TNBC subtypes.

### Validating the prognostic model

TCGA_BRCA dataset was randomly grouped and chosen as a validation dataset to evaluate the prediction efficiency of the risk model. The expression and OS data of prognostic miRNAs were extracted from the validation group. We then calculated the HR and 95% CI of prognostic miRNAs using Cox analysis with a cut-off of *p* < 0.05. Additionally, we constructed a prognostic model based on the validation group. The reliability and validity of the risk model were evaluated using a calibration curve, ROC curve, and KM analysis, respectively.

### Functional enrichment analysis

Based on the validated results, we identified miR-127 as an independent protective prognostic factor for BC. We first predicted the potential target genes by miR-127 using the TargetScan, miRTarBase, and miRNet databases. Common target genes were obtained from the Venn diagrams. We then performed the Gene Ontology (GO) terms [27] and Kyoto Encyclopedia of Gene and Genomes (KEGG) [28] pathway analyses to elucidate the potential function and pathway of miR-127 in cancer. GO terms and KEGG pathways were performed using the DAVID [29] and KO-Based Annotation System (KOBAS) databases [30] separately. Benjamini and Hochberg’s method was used to calculate *p*-values. And the top 10 results were visualized using “ggplot2” package [31]. Additionally, we detected the functional enrichment of miR-127 in hallmark gene sets by Gene Set Enrichment Analysis (GSEA) analysis. We ranked GSEA results with NES (normal enrichment score) and depicted the top 5 items using "ggplot2" package.

### Quantitative real-time PCR assay

The breast cancer cell lines MDA-MB-231, MCF7, SKBR3, and BT474, and the normal breast epithelial cell line MCF-10A were purchased from the cell bank of the Chinese Academy of Sciences. MDA-MB-231, MCF7, and MCF-10A cells were cultured in Dulbecco’s modified Eagle’s medium (DMEM) supplemented with 10% fetal bovine serum (FBS). BT474 cells were cultured in RPMI-1640 medium supplemented with 10% fetal bovine serum (FBS). Cells were maintained at 37 °C in an incubator with 5% CO_2_.

Tumor and adjacent non-tumorous (normal) tissues were collected from 12 patients with BC who underwent surgical treatment at Shengjing Hospital from January 2020 to February 2021. All specimens were collected following surgical resection and immediately frozen at − 80 °C until use. All patients provided written informed consent before enrollment, and the study was approved by the ethics committee of Shengjing Hospital of China Medical University (2021PS569K).

Tissue and cell proteins were lysed using TRIzol reagent to extract the total RNA. For quantitative real-time PCR, NanoPhotometer 50 (Implen, Germany) was used to detect the concentration and purity of total RNA, and a two-step method was then used to synthesize cDNA using a miRNA reverse transcriptase kit. The reaction conditions were as follows: 42 °C for 2 min, 25 °C for 5 min, 50 °C for 15 min, and 85 °C for 5 min, followed by cooling at 4 °C. The amplification reaction was performed using an ABI 7500 system (Thermo Fisher, USA). The amplification system consisted of 2μl cDNA, 0.4 μl mQ primer, 2 μl Specific primer, and 10 μl miRNA universal SYBR, with the total reaction volume adjusted to 20 μl using ddH_2_O. The amplification conditions were as follows: denaturation at 95 °C for 30 s, followed by one cycle of 95 °C for 5 min, 40 cycles of 95 °C for 10 s and 60 °C for 30 s, and one cycle of 95 °C for 15 s, 60 °C for 60 s, and 95 °C for 15 s. U6 was used as an endogenous reference. Primer specificity was determined using melting curve analysis, and relative quantification was performed using the 2 − ΔΔCt method. The primers were synthesized by Shengong Company, and the sequences were as follows: hsa-miR-127-5p (miR-127): 5′-CGC TGA AGC TCA GAG GGC-3′ (forward), 5′-AGT GCA GGG TCC GAG GTA TT-3′ (reverse); miR-127RT Primer: GTC GTA TCC AGT GCA GGG TCC GAG GTA TTC GCA CTG GAT ACG ACA TCA GA; U6:5′-CGC AAG GAT GAC ACG CAA AT-3′ (forward), 5′-CGG CAA TTG CAC TGG ATA CG-3′(reverse).

### Cell transfection

Agomirs and antagomirs of miR-127 (agomiR-127 and antagomiR-127) were designed and synthesized by GenePharma Company (Shanghai, China). Cells were transfected according to the manufacturer's instructions. The transfection procedures were as follows: cells were inoculated in 6-well plates in advance and transfected with Lipofectamine 3000 when the convergence properties reached 70%. Dissolved diluted Lipo 3000 and miR-127 were mixed and allowed to react at room temperature for 20 min before being added to 6-well plates. The changes in miR-127 were detected via PCR to verify the transfection efficiency and conduct subsequent experiments.

### Experimental verificationin vitro

After miR-127 transfection, BC cell proliferation, invasion, and migration were detected using CCK8, transwell, and scratch wound assays, respectively.

The CCK8 kit was used to evaluate changes in cell proliferation after transfection with agomiR-127 and antagomiR-127. The steps followed are as follows: transfected cells during logarithmic growth were incubated in 96-well plates overnight; 10μL of CCK8 solution was added to each well of the plate at 0, 24, 48, and 72 h; the plate was incubated for 1 h in an incubator, and the absorbance was measured at 450 nm using a microplate reader.

A transwell assay was used to assess changes in cell invasion. Matrigel was added to the upper chamber and allowed to solidify in advance. Then, a medium containing 10% FBS was added to the lower chamber as a chemoattractant, and a serum-free medium was added to the upper chamber with matrigel. The cells were seeded in the upper chamber and incubated for 24 h. The lower cells were fixed with 4% paraformaldehyde and stained with hematoxylin and eosin. The number of invading cells in each group was observed under a microscope and statistically analyzed.

A wound-healing assay was performed to assess cell migration. Cells were seeded into 6-well plates and scratched with a 200μL pipette tip when cell convergence reached 70%. The change in the cell scratch width was observed under a microscope at 0 and 24 h and the change in cell mobility was estimated.

### Xenograft tumors formation

The experimental protocol was approved by the ethics committee, Shengjing Hospital (2021PS569K). Female BALB/c nude mice at 8 weeks old were purchased from Beijing HFk Bioscience (Beijing, China), and housed in a specific pathogen-free facility. For Xenograft tumor formation, 1 × 10^6^ cells were subcutaneously injected into the left axillary of each mice. Tumor volume (V) was measured at 7, 14, and 21 days as the following method, V = π/6 × L × W^2^. Finally, all the mice were euthanized with CO_2_ anesthesia and dissected for further analysis. Tumors were weighed and pictured for further analysis.

### Statistical analysis

All statistical analyses were performed using GraphPad Prism 8.0, and R software. For statistical methods, a *t*-test was used to compare significant differences between two groups, and one-way analysis of variance ANOVA to compare multiple groups. Cumulative survival was calculated using Kaplan–Meier univariate analysis. The log-rank test was performed to test for differences in survival time. All experiments were repeated three times, and statistical significance was set at *p* < 0.05 (*p < 0.05).

## Results

### Identification of DEMs

**Fig. 1:**
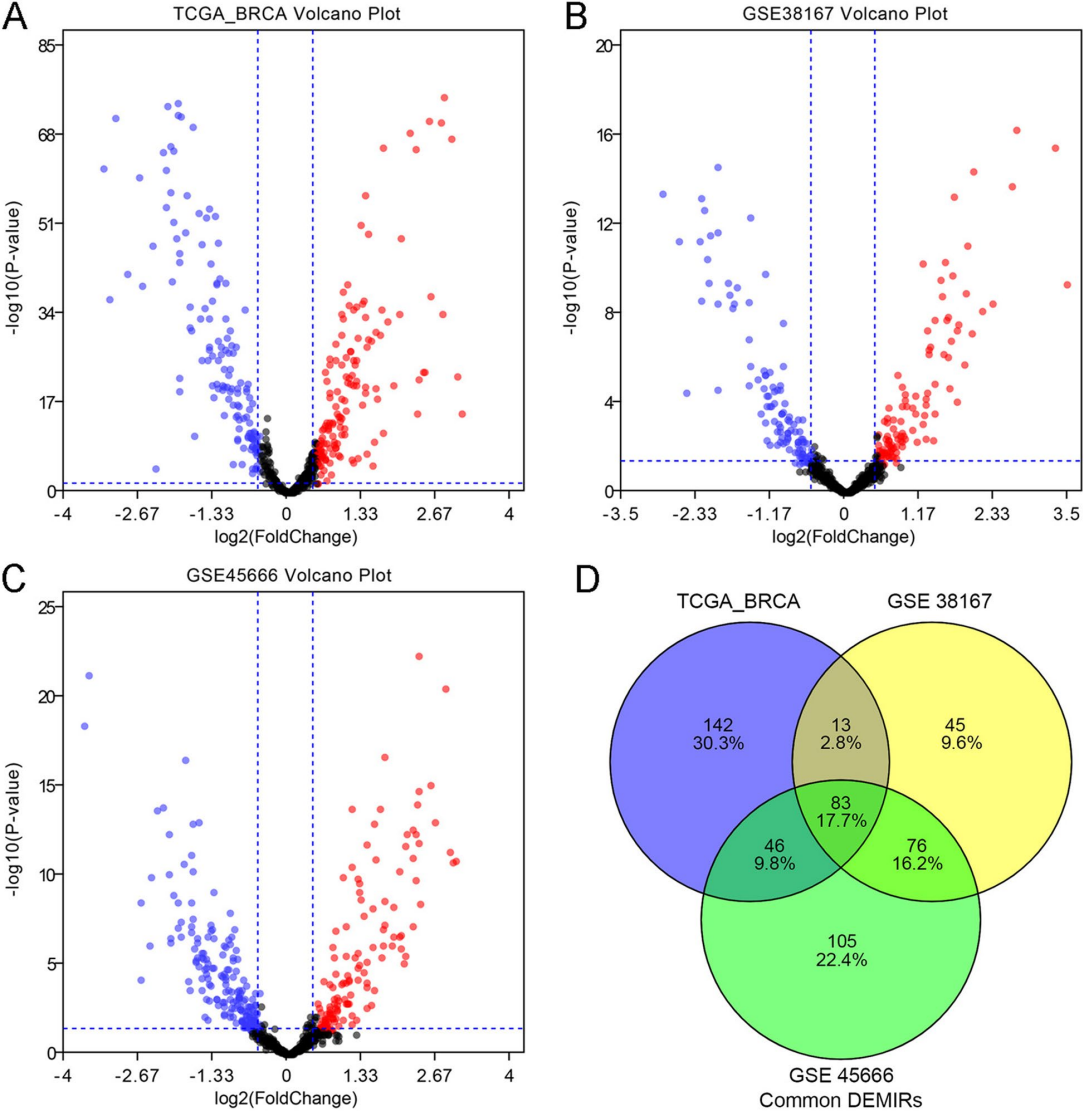
Screening differentially expressed miRNAs (DEMs)between breast cancer(BC) and normal breast. **A**-**C** Volcano plot revealed DEMs in TCGA_BRCA (**A**), GSE38167 (**B**), GSE45666 (**C**), with the threshold of |log2FC|≥ 0.5, *P*-value < 0.05.The red dots and blue dots represent the up-regulated and down-regulated DEMs, respectively. Black means no difference in expression; **D** Venn diagram showed the intersection of DEMs in three groups

A total of 1400 samples were used: 1249 BC samples and 151 normal breast samples from three datasets. DEMs were identified with the threshold of |log2FC|≥ 0.5, *P*-value < 0.05, and displayed on the volcano plot (Fig. 1A-C). Briefly, the TCGA-BRCA database included 284 common DEMs, including 146 upregulated and 138 downregulated DEMs; 217 DEMs in the GSE38167 database, including105 up-regulated and 112 downregulated DEMs; and 323 DEMs in the GSE45666 database, including 137 upregulated and 186 downregulated DEMs. Ultimately, we identified 83 common DEMs, including 44 upregulated and 39 downregulated DEMs (Fig. 1D). The common DEMs in the three datasets were chosen for further analysis.

### Constructing a prognostic model

**Fig. 2:**
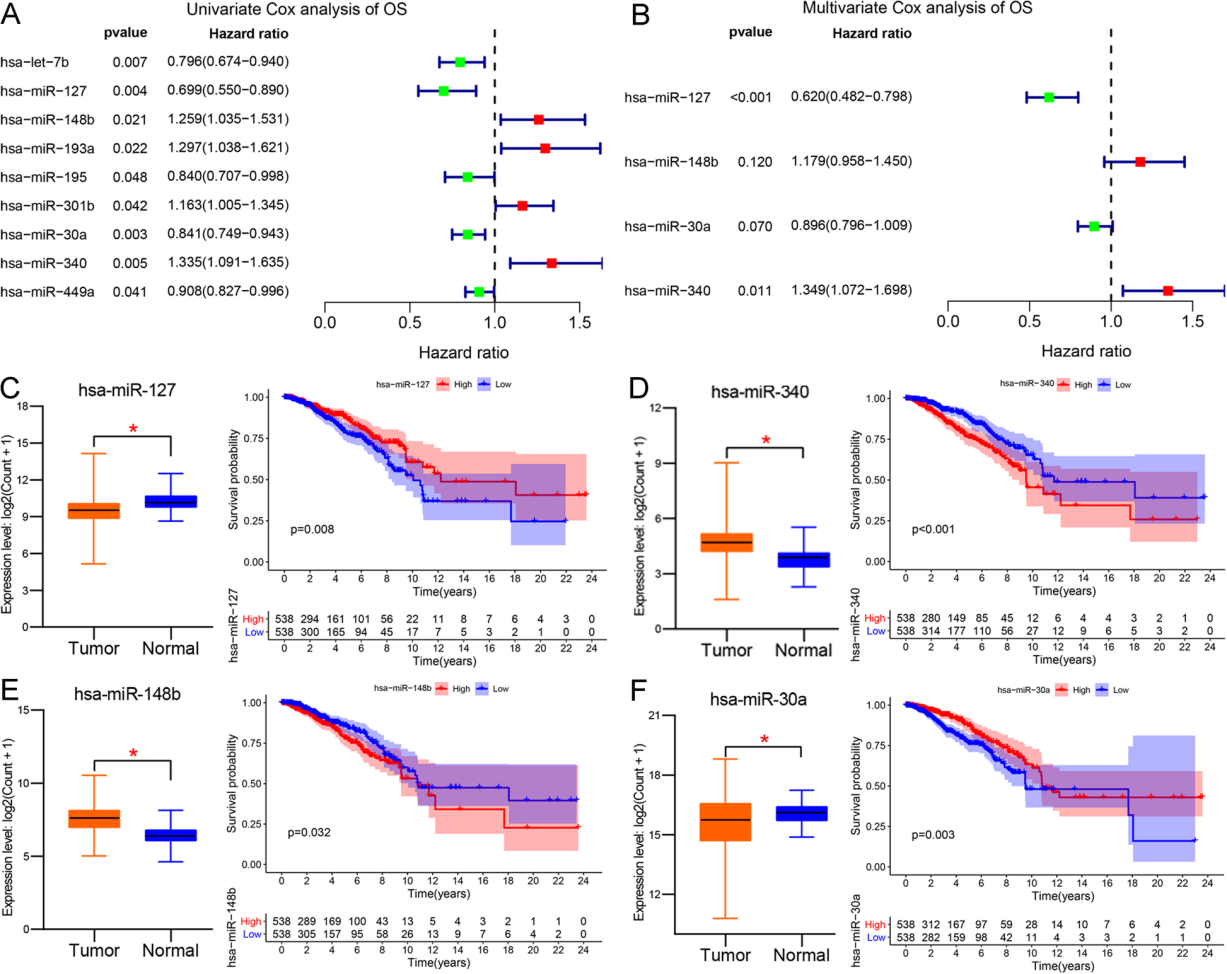
Identification and validation of prognostic DEMs in BC. **A**, **B** Univariate Cox analysis evaluated the prognostic effect of common DEMs (**A**); multivariate Cox analysis was applied to further verify (**B**); HR > 1 was a risk factor, HR < 1 was a protective factor, and *P* < 0.05 was chosen as the screening threshold. **C-F** TCGA_BRCA database verified the expression of hsa-miR-127, hsa-miR-340, hsa-miR-148, and hsa-miR-30a in breast cancer and its relationship with prognosis, with *P* < 0.05.The asterisk (*) means *p*-value < 0.05

**Fig. 3:**
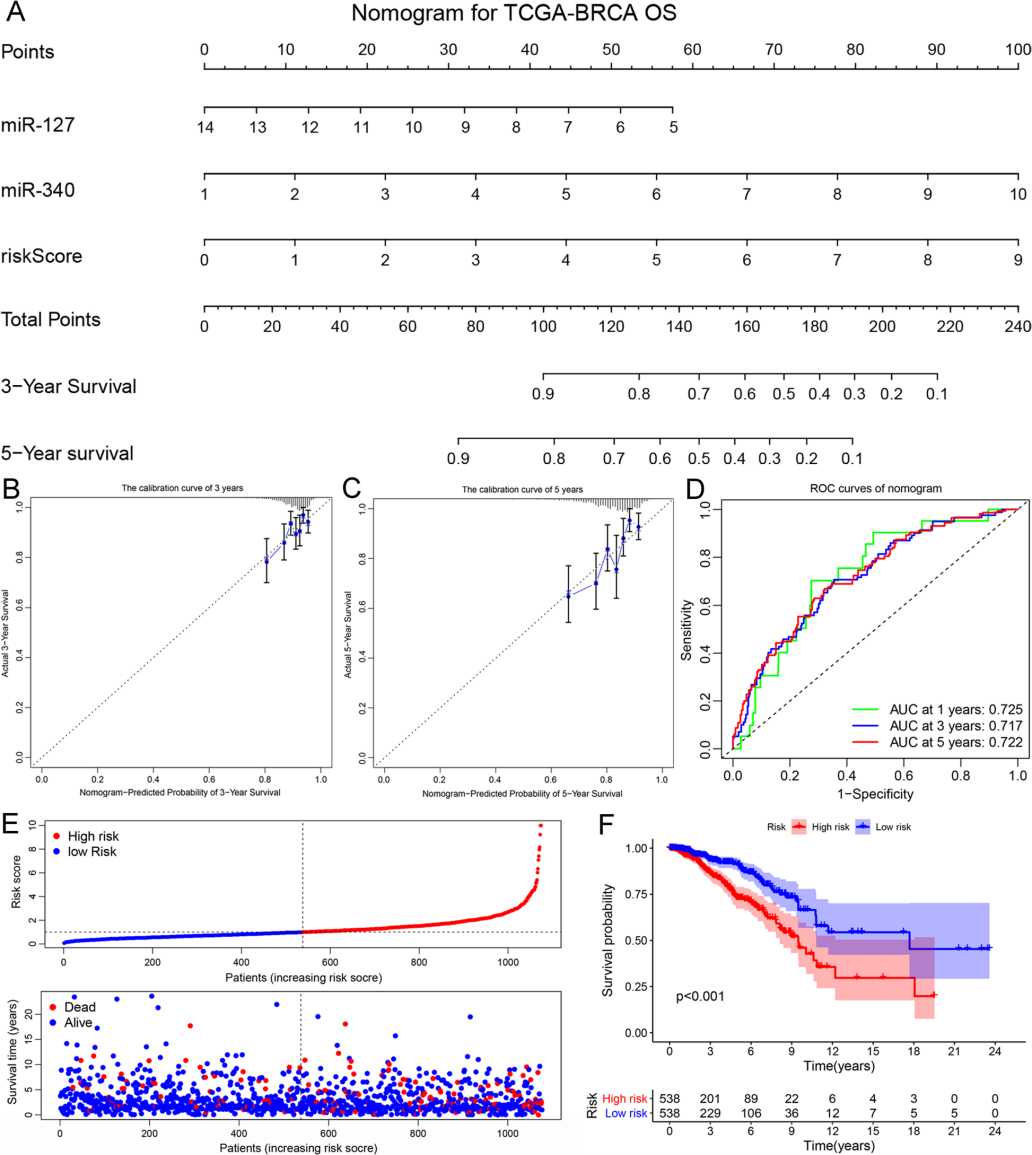
Construction and validation of a nomogram risk model based on prognostic DEMs. **A** Establishing a nomogram risk model in the TCGA_BRCA database; **B**, **C** Calibration curves evaluated the reliability of nomogram for predicting BC 3-year and 5-year overall survival (OS); **D** ROC curves were applied to compare the sensitivities and specificities of the risk model in 1, 3, and 5-year OS; **E** the distribution of risk scores and OS status in dot-plot (**F**); KM survival analysis assessed OS differences in high-risk and low-risk patients

We evaluated the roles of 83 DEMs on the OS of BC through univariate and multivariate Cox analyses and Lasson analysis (Fig. 2A-B and S 1A-B). As presented in Fig. 2A, nine DEMs were associated with BC prognosis as per univariate Cox analysis. We also validated their expression and prognosis in the TCGA_BRCA database (Fig. 2C-F and Figure S 1C-F). The expression of only seven miRNAs was consistent with the univariate Cox analysis. The expression of miR-193a and miR-449a was inconsistent with the results of the univariate analysis (Figure S 1C). The expression and prognosis of the other seven DEMs were matched to univariate analysis with a cut-off threshold of *P* < 0.05 (Fig. 2C-F and S 1C-F). Then, Lasson regression was then performed to determine the best variables included in the multivariate analysis. The results showed that seven prognostic DEMs were suitable for enrollment in multivariate analysis (Figure S 1A-B). In further multivariate Cox analysis, only two DEMs were identified as prognostic miRNAs of BC with a *P*-value of < 0.05, including hsa-miR-127 and hsa-miR-340. The HR and 95%CI of miR-127 and miR-340 were 0.650(0.506 − 0.835), with *P* < 0.001, and 1.498(1.214 − 1.849), with *p* < 0.001, respectively. The expression and prognosis of miR-127 and miR-340 were consistent with the multivariate Cox analysis (Fig. 2C-D). Based on the multivariate analysis, we calculated the prognostic risk scores of each patient. The patients were divided into high-risk and low-risk groups based on the median risk score. The risk formula was as follows: (0.40 × miR-340) − (0.43 × miR-127). A nomogram risk model was constructed with high accuracy to predict the prognostic risk of patients with BC at 3 and 5y (Fig. 3A). Calibration curves revealed that the prognostic prediction of the nomogram at 3-year and 5-year was consistent with the actual OS (Fig. 3B-C). Additionally, we depicted the distribution of the risk scores and OS status in a dot-plot. The results showed a substantial increase in mortality with increasing risk scores (Fig. 3E). The ability of the nomogram to predict OS was evaluated using the ROC curve and KM survival analysis (Fig. 3D-F). The ROC curve showed that the nomogram was excellent for predicting 1-, 3-, and 5-year OS with AUC values of 0.73, 0.72, and 0.72, respectively (Fig. 3D). Survival analysis showed that the high-risk group had a worse prognosis and higher mortality than the low-risk group (Fig. 3F). These results indicated that the prognostic risk model constructed based on DEMs could effectively predict BC OS. Furthermore, we validated the relationship between miR-127-5p and clinical paraments in the METABRIC database (Figure S 2 A-F). High expression of hsa-miR-127-5p indicated a better prognosis in overall survival, ER-positive group, HER2 negative group, TNBC, lymph node positive group, and luminal A subtype. Those findings reveal that miR-127 research has a promising clinical application for BC therapy. Also, we revealed the characteristic of hsa-miR-127 and hsa-miR-340 in PAM50 subtypes. As shown in Figure S 3 A-B, the expression of miR-340 in LumA (Luminal A) was lower than in LumB, Basel, and Normal (TNBC) subtype. The survival of LumA subtype is better than other PAM50 subtypes, and miR-340 has a higher expression in the TNBC subtype. High expression of miR-340 reveals a worse survival, which is consistent with our previous results (Fig. 2D). Similarly, miR-340 in LumA (Luminal A) was higher than in LumB, Basel, and Normal (TNBC) subtype. And the high expression of miR-340 reveals a better survival, which is consistent with our previous results (Fig. 2C).

### Validating the prognostic model

**Fig. 4:**
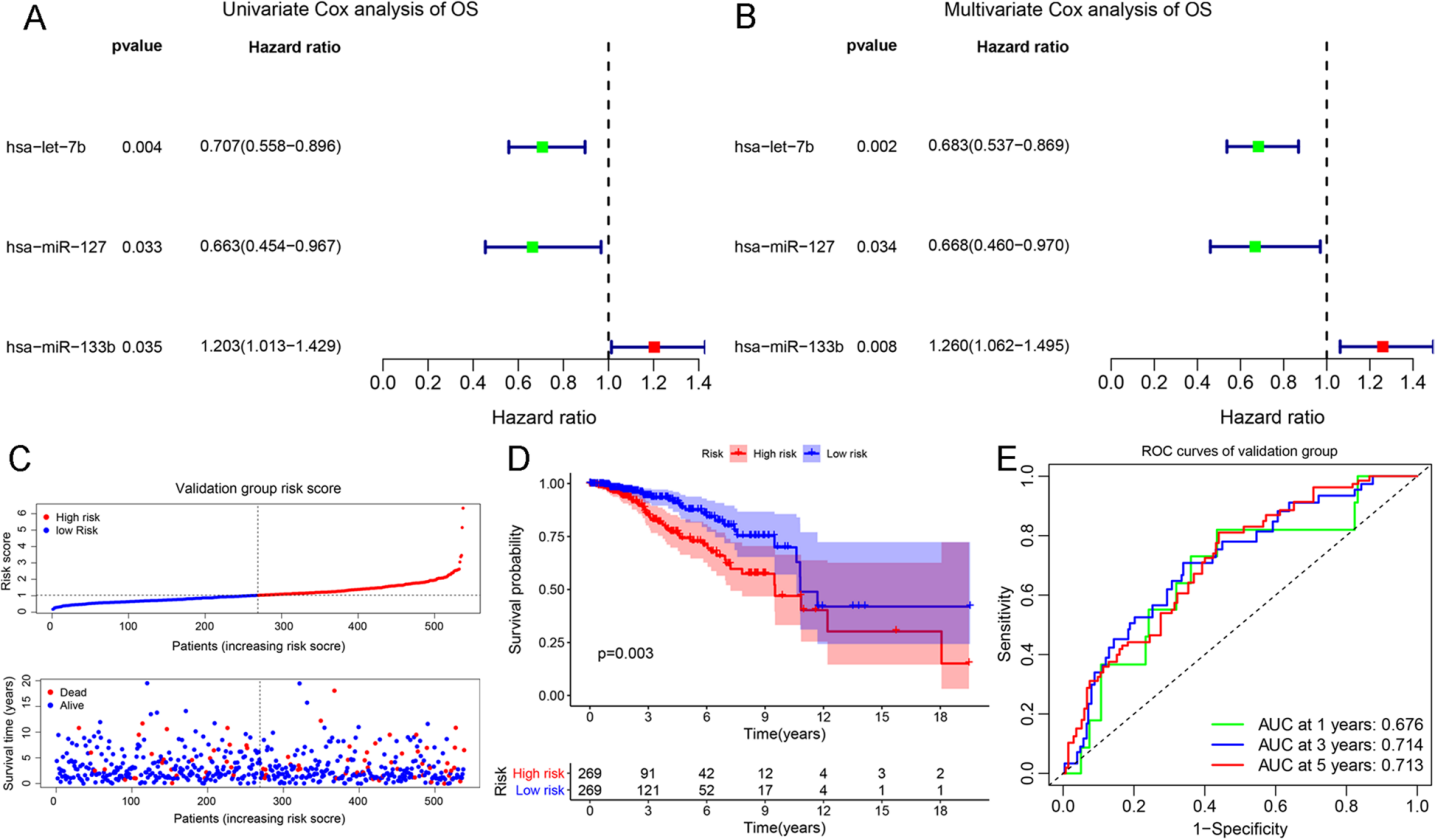
Evaluating the prediction performance of the risk model. **A, B** Univariate and multivariate Cox analyses were performed to verify the effect of DEMs on OS; HR > 1 was a risk factor, HR < 1 was a protective factor, and *P* < 0.05 was chosen as the screening threshold; **C-E** Validating the prognostic prediction ability of risk model; the distribution of OS status in high-risk and low-risk breast cancer patients were presented in dot-plot (**C**); KM survival analysis assessed OS differences in high-risk and low-risk patients (**D**); ROC curves was applied to compare the sensitivities and specificities of BC risk model in 1, 3, and 5 years (**E**)

In the validation group, we further evaluated the relationship between the DEMs and BC survival outcomes. Three prognostic miRNAs were identified via univariate and multivariate Cox analysis, named hsa-let-7b, hsa-miR-127, and hsa-miR-133b (Fig. 4A-B). Briefly, the HR and 95% CI of let-7b, miR-127, and miR-133b in the multivariate analysis were 0.620(0.482 − 0.798) with a *P*-value of 0.002, 0.668(0.460 − 0.970) with a *P*-value of 0.034, and 1.260(1.062 − 1.495) with a *P*-value of 0.008, respectively. Furthermore, we constructed a nomogram risk model and tested its ability to predict prognosis. The risk formula is as follows: (0.23 × miR-133b) − (0.40 × miR-127) − (0.38 × let-7b). Based on the dot-plot, we found that patient mortality increased markedly in the high-risk group (Fig. 4C). Survival analysis showed that the high-risk group had a worse prognosis than that of the low-risk group (Fig. 4D). The ROC curve showed that the ability of the model to predict the 1-year, 3-year, and 5-year OS rates of BCwere 0.68, 0.71, and 0.71, respectively (Fig. 4E). According to previous results, we confirmed miR-127 as an independent prognostic miRNA and were chosen for cell line and tissue analysis. To further discuss the treatment strategies of miR-127 in BC, we compared its expression difference in endocrine therapy (ER/PR) and targeted therapy (HER2). As shown in Figure S 4, miR-127 shares a lower expression in ER, PR, HER2 positive status, PR, and HER2 high positive status. Those findings indicated the exogenous supplement of miR-127 could have a clinical treatment response in patients with PR and HER2 positive expression. These results reveal that miR-127 can be regarded as a potential target for BC therapy.

### Functional enrichment results

We confirmed that low levels of miR-127 are an independent protective factor for BC. Next, we performed GO term and KEGG analyses to explore the potential biological functions of miR-127 and the pathways involved. Target genes of miR-127 were also predicted. As shown in Figure S 5, 3002 target genes were found in the TargetScan database, 588 target genes in the miRTarBase database, and 269 target genes in the miRNet database, with 91 common target genes among the three databases. Ninety biological processes and 18 KEGG pathways were enriched by miR-127 expression. The top ten GO terms and KEGG pathways are shown in the barplot (Figure S 5B-C). Briefly, miR-127 is mainly related to these biological processes, including protein binding, zinc ion binding, and RNA polymerase transcription, as well as several pathways, such as the PI3K-Akt, p53, and microRNA cancer pathways. In GSEA analysis, the top five GO and KEGG terms are shown in Figure S 5E-F. Our results share a similar function between GSEA and KEGG and GO analysis such as extracellular exosome and p53 signaling pathway, which indicated the reliability of our results. Overall, these results suggest that miR-127 is closely involved in the biological processes and pathways of cancer.

### miR-127 regulating BC proliferation, migration, and invasion

**Fig. 5:**
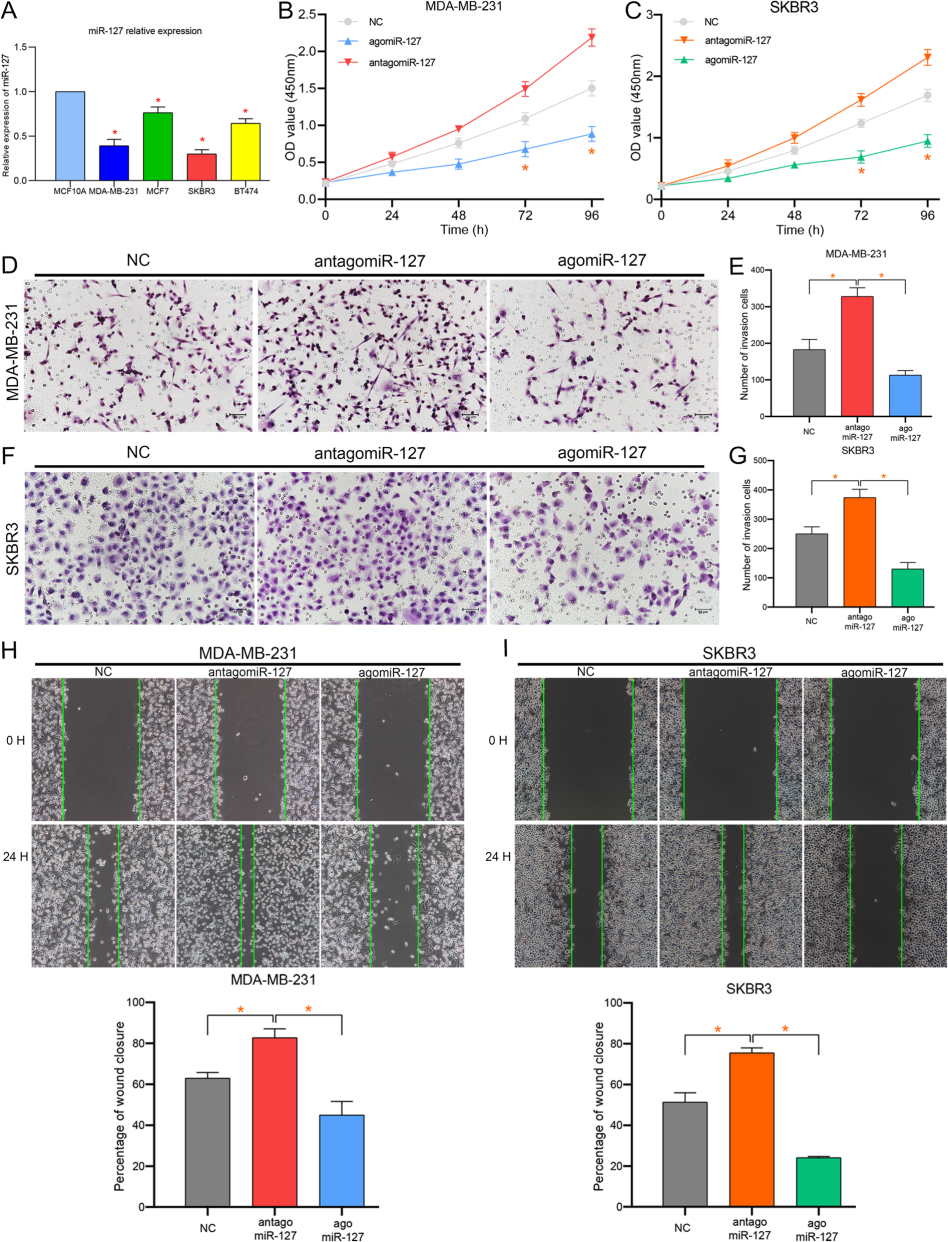
Low expression of miR-127 promotes BC proliferation, invasion, and migration. **A** The relative expression of miR-127 was evaluated in breast cancer cells and normal breast cells; **B-I** Cell proliferation, invasion, and migration were detected via CCK8, Transwell, and scratch wound assays, respectively. the interference or overexpression of miR-127 significantly enhanced or inhibited the proliferation of SKBR3 and MDA-MB-231 cells (**B**, **C**); invasion ability of SKBR3 and MDA-MB-231 cells (**D-G**); cell migration in SKBR3 and MDA-MB-231 (**H**, **I**). (*) means *p*-value < 0.05

After multiple validations and evaluations, we identified miR-127 as an independent prognostic protective factor for BC. Next, we verified the expression of miR-127 in tissues and its effect on BC proliferation, invasion, and migration. The expression of miR-127 in 12 pairs of fresh BC and normal tissues was detected using qPCR. The results showed that miR-127 expression was low in BC (Figure S 5C), which is consistent with our validation results. In addition, the expression of miR-127 in BC cells was remarkably lower than that in normal breast cells. miR-127 expression in SKBR3 and MDA-MB-231 cells was lower than that in BT474 and MCF7 cells (Fig. 5A). SKBR3 and MDA-MB-231 cells were transfected with agomiR-127 and antagomiR-127, respectively. Cell proliferation, invasion, and migration were detected via CCK8, transwell, and scratch wound assays, respectively (Fig. 5B-I). According to the results of the CCK8 assay, the cell activities of SKBR3 and MDA-MB-231 at 72 h and 96 h were markedly promoted following miR-127 interference. In contrast, miR-127 overexpression notably inhibited the activity of SKBR3 and MDA-MB-231 (Fig. 5B-C). Moreover, the ability of SKBR3 and MDA-MB-231 cells to cross the Matrigel could be substantially increased through miR-127 silencing and decreased through miR-127 overexpression (Fig. 5D-G). In cloning experiments, the healing ability of SKBR3 and MDA-MB-231 cells was considerably increased and decreased after miR-127 silencing and overexpression, respectively (Fig. 5H-I). These results suggest that low expression levels of miR-127 promoted BC proliferation, migration, and invasion.

### miR-127 promotes tumor growthin vivo

**Fig. 6:**
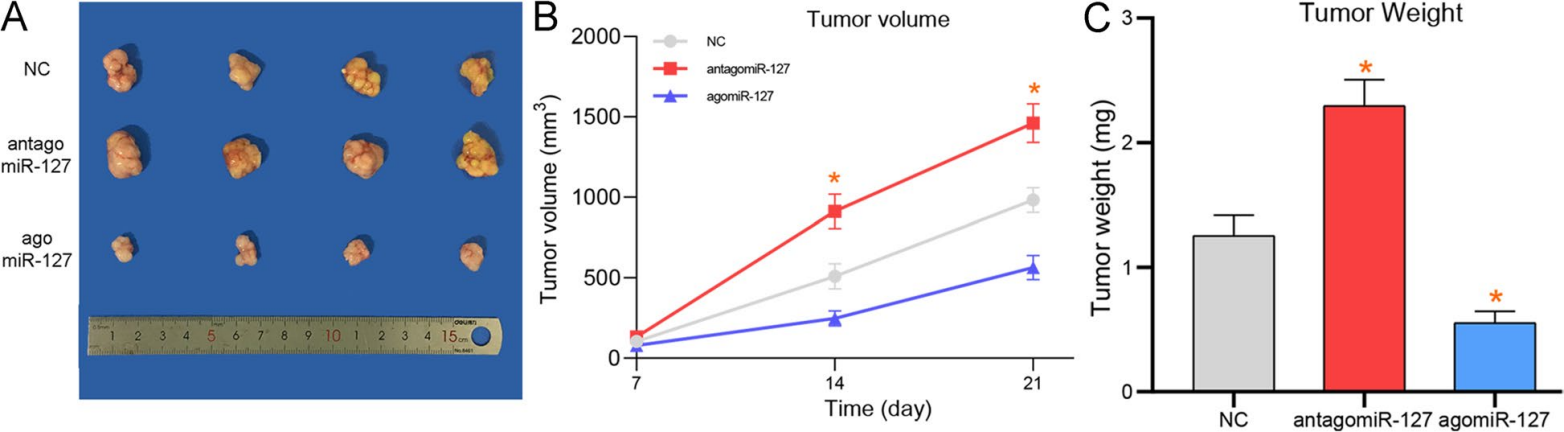
MiR-127 regulates tumor growth in vivo. **A**, **B** Tumor volume was significantly up-regulated and down-regulated after miR-127 interference and overexpression. **C** Tumor weight was increased or decreased in mice after miR-127 silence and overexpression. (*) means *p*-value < 0.05

To confirm the potential role of miR-127 in vivo, we established xenograft tumors in BALB/c nude mice. Nude mice were injected with MDA-MB-231 cells in the left axilla and were randomly divided into three groups, including NC, antagomiR-127, and agomiR-127. We assessed the changes in tumor formation, volume, and weight. As shown in Fig. 6A-B, tumor volume was substantially increased and decreased following antagomiR-127 and agomiR-127 transfection, respectively. Meanwhile, tumor weight was markedly increased and decreased following miR-127 silencing and overexpression, respectively (Fig. 6C). These results suggest that miR-127 regulates breast cancer growth and progression in vivo and is a potential target for breast cancer treatment.

## Discussion

The dysregulation of miRNAs plays a major role in cancer formation, progression, and metastasis because miRNAs are powerful regulators of mRNAs [32]. On the one hand, tumor suppressor miRNAs mediate the degradation of specific oncogenes and inhibit the occurrence, proliferation, apoptosis, and invasion of tumors [33]. On the other hand, oncogenic miRNAs regulate the normal expression of tumor suppressor genes, causing homeostasis imbalance and promoting tumor progression [34]. Moreover, miRNAs regulate the translation level of downstream genes without changing the transcription of mRNA, which is valuable for developing new drugs [35]. In addition, miRNAs are extensively involved in BC proliferation, cell cycle progression, tumor invasion, angiogenesis, and metastasis [36]. Therefore, it is necessary to elucidate the underlying mechanisms of miRNAs in BC.

In this study, we summarized the roles of miRNAs in BC and revealed their regulatory mechanisms. Using multiple miRNA databases, we comprehensively explored the expression of miRNAs in BC and established and validated a miRNA-based prognostic model. The ROC curve and KM analysis suggested that the prognostic model has a good predictive ability for OS. We identified miR-127 as an independent prognostic factor for OS. Hsa-miR-127 is in the chromosome region 14q32.2, with the sequence CUG AAG CUC AGA GGG CUC UGAU [37, 38]. The transcription of miR-127 is induced by estrogen-related receptor gamma (ERRc) and inhibited by a small heterodimer partner (SHP) [39, 40]. MiR-127 has been shown to play a tumor suppressor role in a variety of human cancers, including BC [41]. Hypermethylation of the miR-127 promoter region in BC tissues is strongly associated with metastasis and is a marker of tumor metastasis [42]. MiR-127 was substantially downregulated in BC tissues. The low expression level of miR-127 is associated with lymph node metastasis, clinical stages, and shorter OS, which are independent factors for BC prognosis [43]. In BC cells, the oncogene BCL6 [44] and cancer progression-related genes ROCK2 and CDH11 [45] have been identified as targets of miR-127 in BC. Low miR-127 expression promotes tumor progression by increasing oncogene expression. In triple-negative BC, miR-127 overexpression increases stem cell sensitivity to radiotherapy drugs [46]. In addition, miR-127 inhibits the proliferation and invasion of gastric cancer cells via Wnt7a [47], inhibits ovarian cancer cell proliferation by downregulating MAPK4 [48], regulates the NF-κB pathway through TNFAIP3, and induces epithelial-mesenchymal transformation in lung cancer [49]. These findings were consistent with our results. Overall, we comprehensively explored the role of miR-127 in BC expression, prognosis, proliferation, migration, invasion, and tumorigenesis.

Although previous studies have explored the role of miR-127 in BC, this study is the first to comprehensively describe the role of miRNA-127 in BC prognosis, cell function, and tumorigenesis. Our study has some limitations. For instance, we did not explore the effect of pathological classifications on OS because the impact of pathological types on BC prognosis is widely known [35, 50]. This study lacks a large-sample validation of miR-127 expression and prognosis in BC. However, we obtained results from 1400 samples, which ensured the credibility of our findings. In cell experiments, we validated the roles of miR-127 in proliferation, migration, invasion, and tumorigenesis without exploring the function of its target genes. Therefore, further studies are warranted.

## Conclusions

In conclusion, our study comprehensively revealed the prognostic impact of miRNAs and identified miRNA-127 as an independent protective factor for BC. In vitro and in vivo experiments showed that miR-127affects BC cell expression, proliferation, migration, invasion, and tumorigenesis. These results reveal that miR-127 contributes to BC progression, indicating a potential target for breast cancer treatment.

## Supplementary Information

**Additional file 1:** **Figure S1.** Identification and validation of prognostic DEMs. (A-B) Lasson analysis evaluated the optimal variables of the multivariate Cox regression analysis; (C)TCGA_BRCA database indicated that the expression of hsa-miR-193a and hsa-miR-449a was inconsistent with the univariate results; (D-F)TCGA_BRCA database verified the expression and prognosis of DEMs including hsa-let-7b, hsa-miR-195 and hsa-miR-301b with the *P* <0.05. The asterisk (*)means *p*-value < 0.05.

**Additional file 2:** **Figure S2. **Survival analysis for miR-127 in METABRIC data. (A-F) high expression of miR-127-5p indicated a better prognosis in overall survival (A), ER-positive group (B), HER2 negative group (C), TNBC group (D), lymph node-positive group (E), and luminalA subtype (F).

**Additional file 3:** **Figure S3.** The different expression of miR-340 and miR-127 in PAM50 subgroups. (A, C) The expression of miR-340 (A) and miR-127 (C) in 5 PAM50 subgroups. (B, D) The expression of miR-340 (B) and miR-127 (D) in TNBC, Her2, LumA, and LumA subgroups. (*) means *p* value < 0.05.

**Additional file 4: Figure S4.** The expression characteristic of miR-127 in ER status, PR status, and HER2 status. (A,C,E) miR-127 expression difference in the negative and positive status of ER, PR and HER2 IHC testing, respectively. (B, D, F) the miR-127 difference in ER, PR, and HER2 low expression and high expression separately. (*) means *p*-value < 0.05.

**Additional file 5:** **FigureS5. **Functional enrichment analysis for miR-127. (A) Venn diagram shows the potential binding target genes and common target genes of miR-127 in TargetScan, miRTarBase, and miRNet databases, respectively; (B, C) The barplot shows the top10 GO terms (B) and the top10 KEGG pathways (C); GO and KEGG were ranked according to the count value of genes, with *P* <0.05 was chosen as the cut-off value. (D) The expression of miR-127 in BC tissues. (E, F) The GSEA analysis shows the top 5 GO items (B) and the top 5 KEGG pathways in hallmark genesets (*) means *p*-value < 0.05.

## Data Availability

The datasets obtained during and/or analyzed within the current study are available in the Cancer Genome Atlas, TCGA-BRCA (https://portal.gdc.cancer.gov/cart), (https://xenabrowser.net/datapages/?dataset=TCGA-BRCA.mirna.tsv&host=https%3A%2F%2Fgdc.xenahubs.net&removeHub=https%3A%2F%2Fxena.treehouse.gi.ucsc.edu%3A443), the Gene Expression Omnibus GSE38167 (https://ftp.ncbi.nlm.nih.gov/geo/series/GSE38nnn/GSE38167/matrix/) and GSE45666 (https://ftp.ncbi.nlm.nih.gov/geo/series/GSE45nnn/GSE45666/matrix/).

## Abbreviations

BC: Breast cancer
OS: Overall survival
mRNA: Messenger RNA
ncRNA: Non-coding RNA
miRNA: MicroRNA
ORF: Open reading framework
RISC: RNA-induced silencing complex
DEM: Differentially expressed miRNA
TCGA: The Cancer Genome Atlas
HR: Hazard ratios
CI: Confidence intervals
GEO: Gene expression omnibus
GDC: Genetic Disease Control
DMEM: Dulbecco's modified Eagle's medium
FBS: Fetal bovine serum; KM: Kaplan–Meier
ROC: Receiver operating characteristic
AUC: Area under the curve
GO: Gene ontology
KEGG: Kyoto encyclopedia of genes and genomes
KOBAS: KO-based annotation system
ERRc: Estrogen-related receptor gamma
SHP: Small heterodimer partner
