# Supplementary figures and images for: Integrated analysis identified prognostic microRNAs in breast cancer
Source: BMC Cancer. 2022 Nov 12;22:1170. doi: 10.1186/s12885-022-10242-x (PMC9652801; doi:10.1186/s12885-022-10242-x)

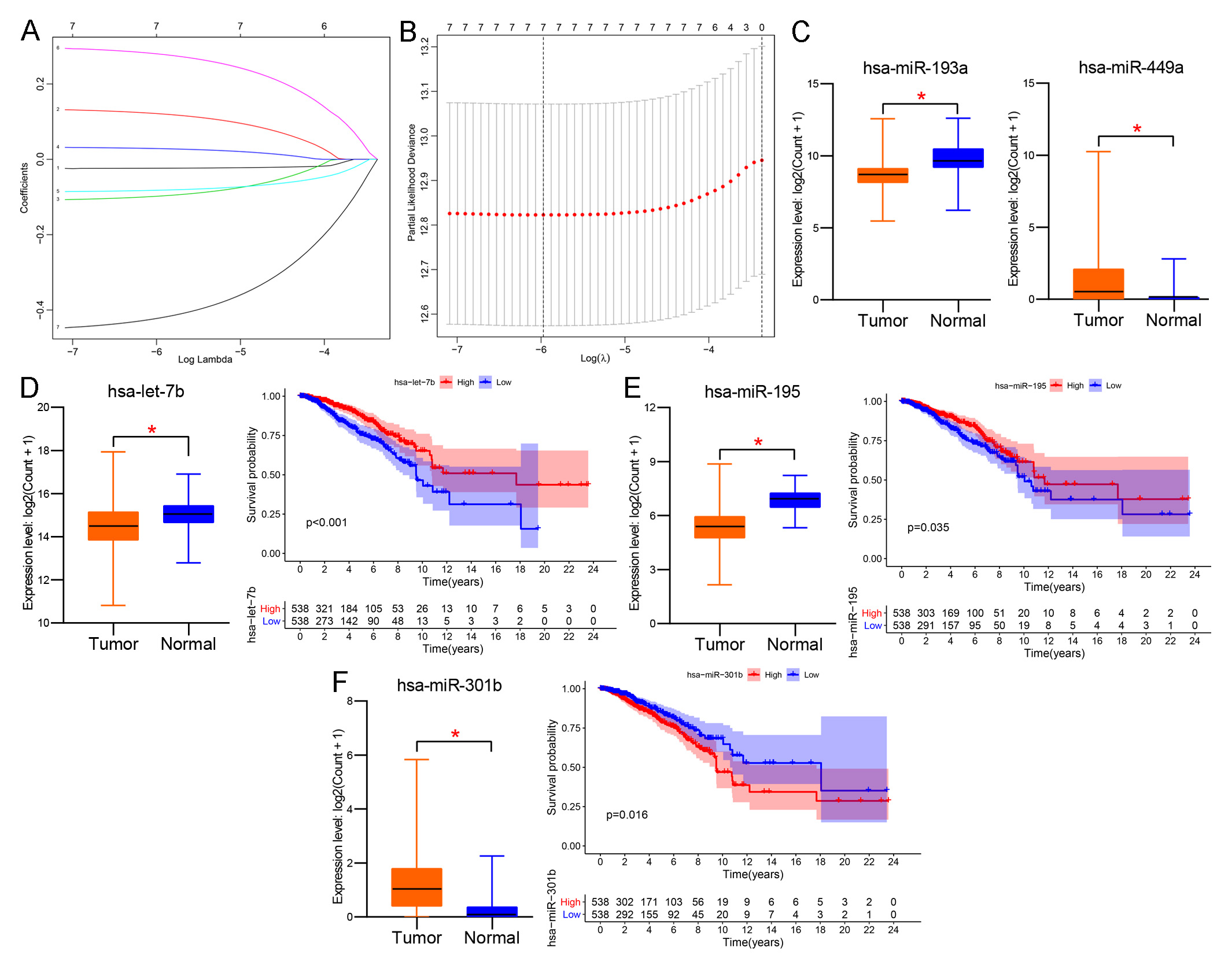

Supplement: Supplementary file 1 — Additional file 1: Figure S1. Identification and validation of prognostic DEMs. (A-B) Lasson analysis evaluated the optimal variables of the multivariate Cox regression analysis; (C)TCGA_BRCA database indicated that the expression of hsa-miR-193a and hsa-miR-449a was inconsistent with the univariate results; (D-F)TCGA_BRCA database verified the expression and prognosis of DEMs including hsa-let-7b, hsa-miR-195 and hsa-miR-301b with the P <0.05. The asterisk (*)means p-value < 0.05. [file 12885_2022_10242_MOESM1_ESM.jpg]

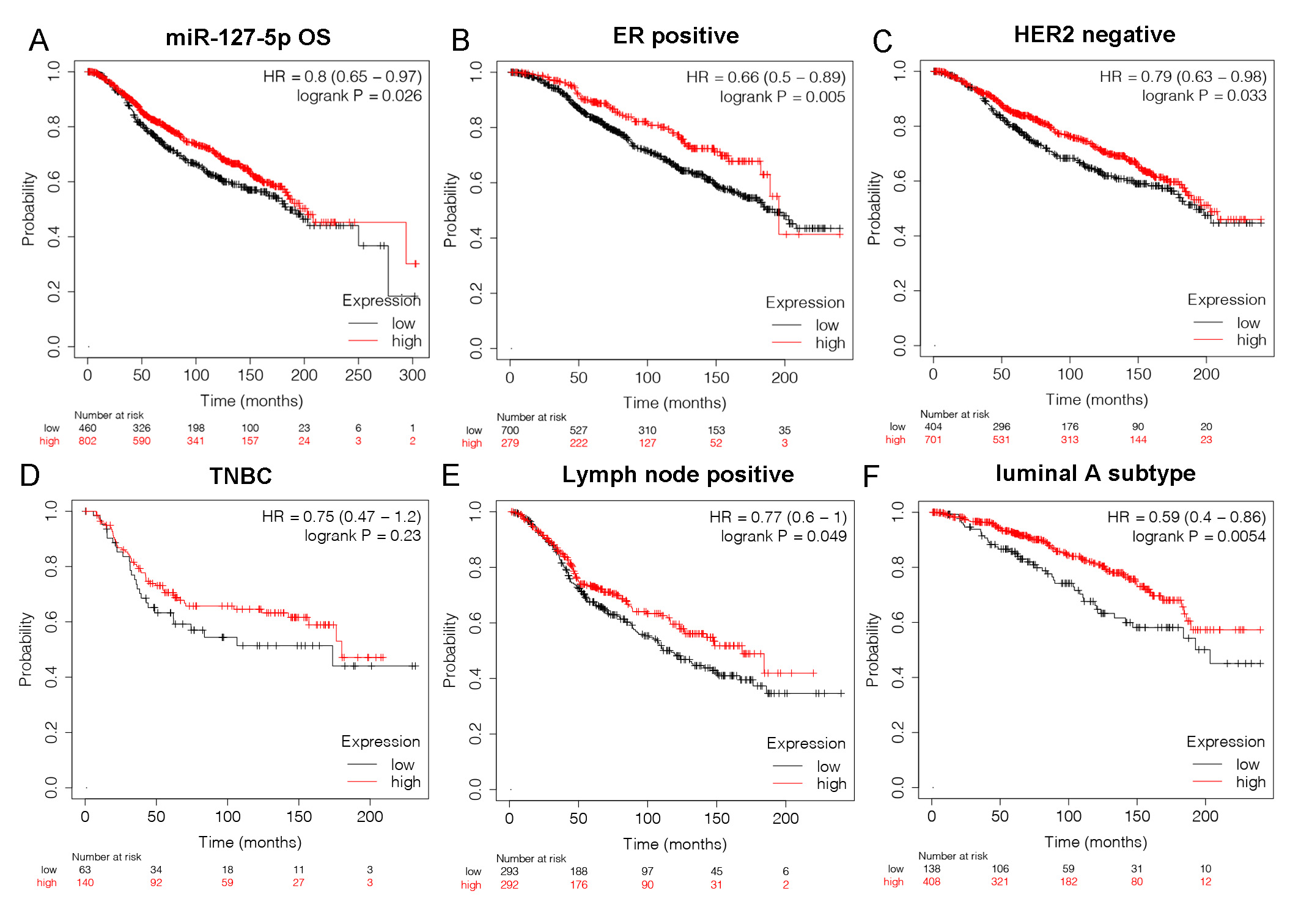

Supplement: Supplementary file 2 — Additional file 2: Figure S2. Survival analysis for miR-127 in METABRIC data. (A-F) high expression of miR-127-5p indicated a better prognosis in overall survival (A), ER-positive group (B), HER2 negative group (C), TNBC group (D), lymph node-positive group (E), and luminalA subtype (F). [file 12885_2022_10242_MOESM2_ESM.jpg]

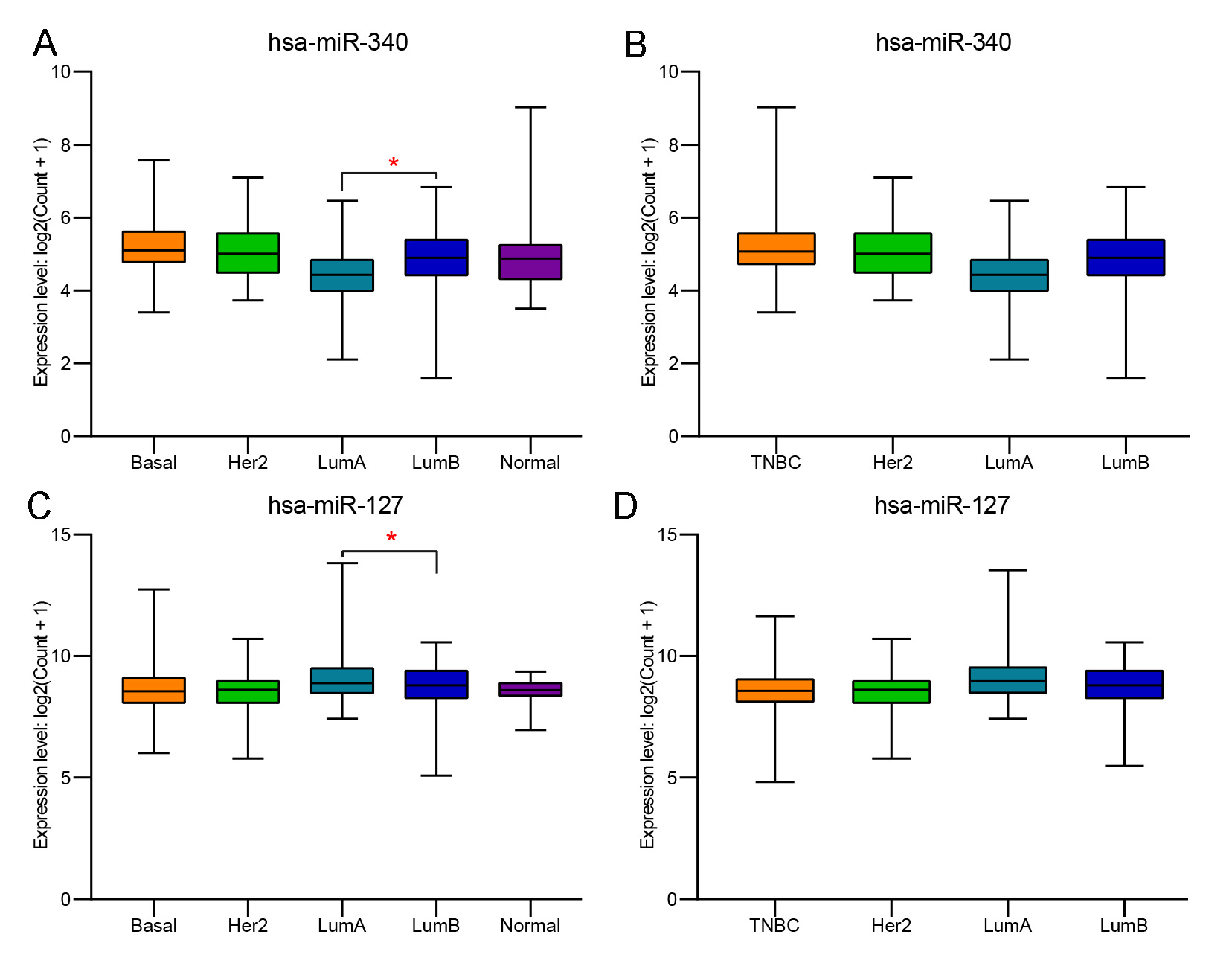

Supplement: Supplementary file 3 — Additional file 3: Figure S3. The different expression of miR-340 and miR-127 in PAM50 subgroups. (A, C) The expression of miR-340 (A) and miR-127 (C) in 5 PAM50 subgroups. (B, D) The expression of miR-340 (B) and miR-127 (D) in TNBC, Her2, LumA, and LumA subgroups. (*) means p value < 0.05. [file 12885_2022_10242_MOESM3_ESM.jpg]

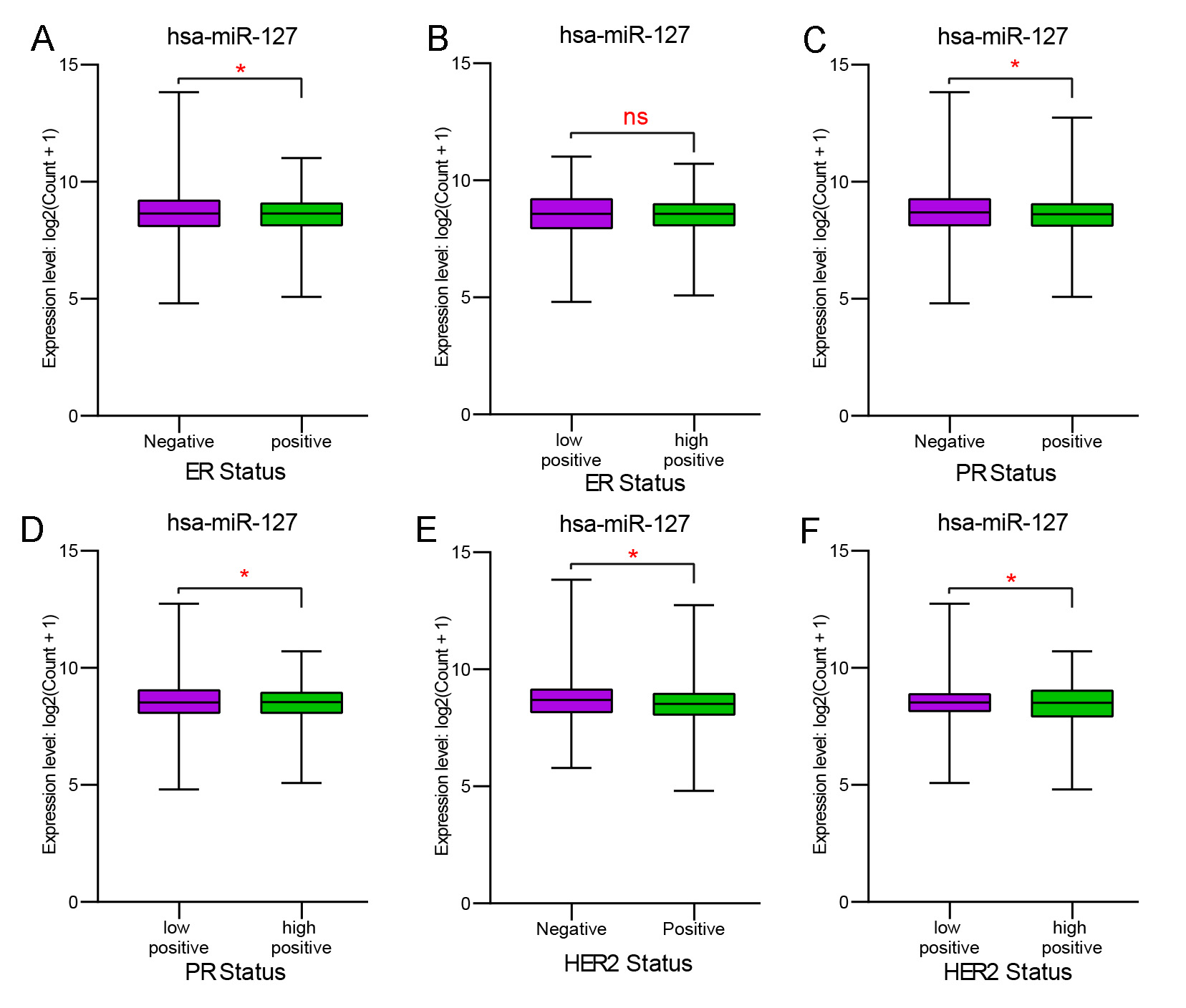

Supplement: Supplementary file 4 — Additional file 4: Figure S4. The expression characteristic of miR-127 in ER status, PR status, and HER2 status. (A,C,E) miR-127 expression difference in the negative and positive status of ER, PR and HER2 IHC testing, respectively. (B, D, F) the miR-127 difference in ER, PR, and HER2 low expression and high expression separately. (*) means p-value < 0.05. [file 12885_2022_10242_MOESM4_ESM.jpg]

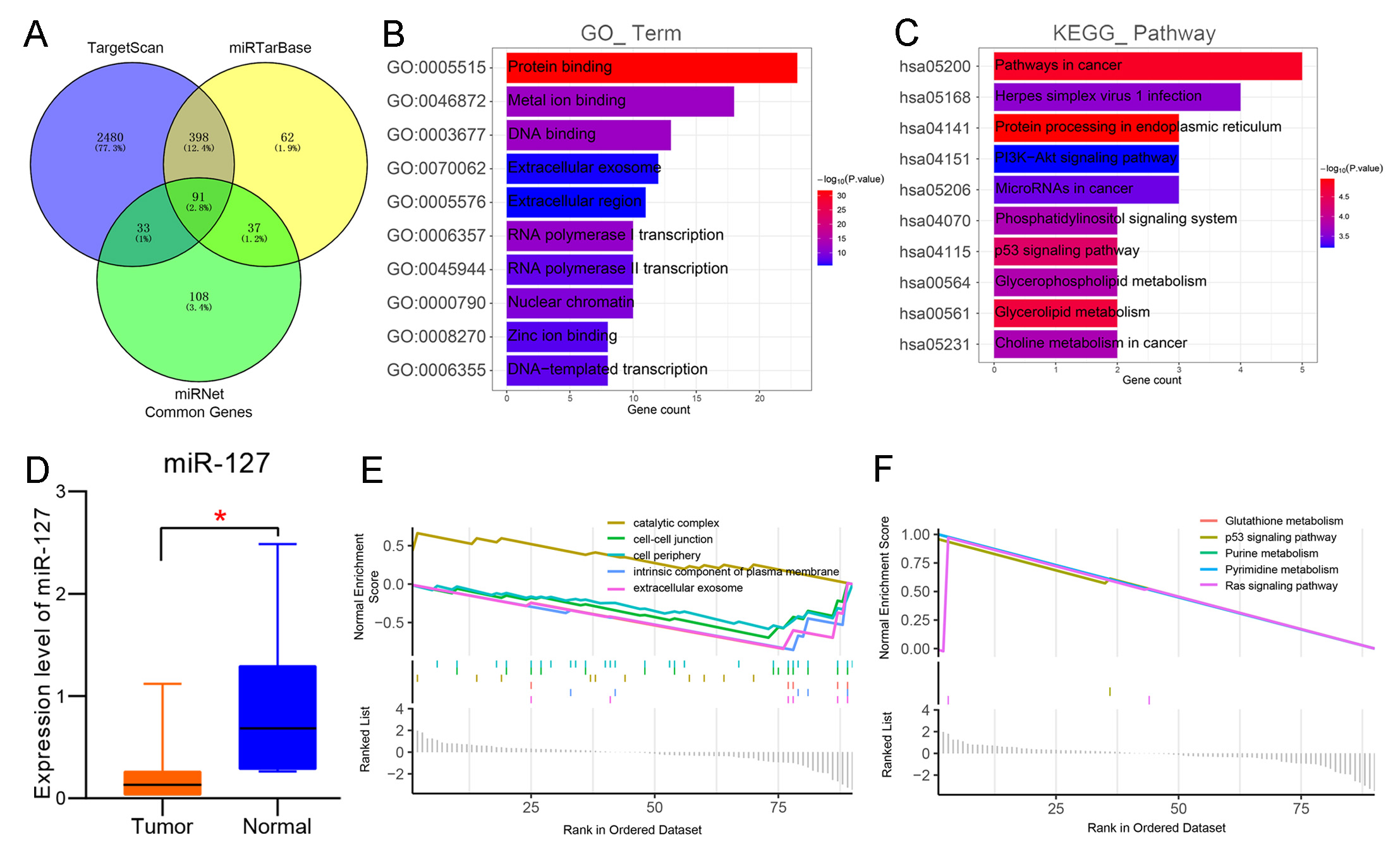

Supplement: Supplementary file 5 — Additional file 5: FigureS5. Functional enrichment analysis for miR-127. (A) Venn diagram shows the potential binding target genes and common target genes of miR-127 in TargetScan, miRTarBase, and miRNet databases, respectively; (B, C) The barplot shows the top10 GO terms (B) and the top10 KEGG pathways (C); GO and KEGG were ranked according to the count value of genes, with P <0.05 was chosen as the cut-off value. (D) The expression of miR-127 in BC tissues. (E, F) The GSEA analysis shows the top 5 GO items (B) and the top 5 KEGG pathways in hallmark genesets (*) means p-value < 0.05. [file 12885_2022_10242_MOESM5_ESM.jpg]
